# Do faces speak volumes? Social expectations in speech comprehension and evaluation across three age groups

**DOI:** 10.1371/journal.pone.0259230

**Published:** 2021-10-28

**Authors:** Adriana Hanulíková

**Affiliations:** 1 Department of German – German Linguistics, Albert-Ludwigs-Universität Freiburg, University of Freiburg, Freiburg, Germany; 2 Freiburg Institute of Advanced Studies (FRIAS), University of Freiburg, Freiburg, Germany; Universite Sorbonne Nouvelle Paris 3, FRANCE

## Abstract

An unresolved issue in social perception concerns the effect of perceived ethnicity on speech processing. Bias-based accounts assume conscious misunderstanding of native speech in the case of a speaker classification as nonnative, resulting in negative ratings and poorer comprehension. In contrast, exemplar models of socially indexed speech perception suggest that such negative effects arise only when a contextual cue to the social identity is misleading, i.e. when ethnicity and speech clash with listeners’ expectations. To address these accounts, and to assess ethnicity effects across different age groups, three non-university populations (N = 172) were primed with photographs of Asian and white European women and asked to repeat and rate utterances spoken in three accents (Korean-accented German, a regional German accent, standard German), all embedded in background noise. In line with exemplar models, repetition accuracy increased when the expected and perceived speech matched, but the effect was limited to the foreign accent, and—at the group level—to teens and older adults. In contrast, Asian speakers received the most negative accent ratings across all accents, consistent with a bias-based view, but group distinctions again came into play here, with the effect most pronounced in older adults, and limited to standard German for teens. Importantly, the effects varied across ages, with younger adults showing no effects of ethnicity in either task. The findings suggest that theoretical contradictions are a consequence of methodological choices, which reflect distinct aspects of social information processing.

## Introduction

The ways in which social attributions to a person’s appearance and voice influence language processing have been a topic of recent debates. A large body of research has provided evidence that personal attributes of a speaker such as gender, ethnicity, regional or linguistic background have a profound effect on language attitudes, speech comprehension, and accent or competence evaluations (e.g., see [1], for a recent review, and [2–13]). These effects are often assumed to reflect an activation of linked social and linguistic information, which can result from learned associations between linguistic patterns and a range of non-linguistic contexts [14] as well as from ideological connections and speaker’s attitudes [15]. For example, [7] has shown that listeners make judgments about who might say what based on the ethnicity of the speaker, as conveyed by photographs of black and white individuals. In her study, listeners related the likelihood of /t/-deletion in a word such as *mast* to black ethnicity and responded faster to sentence continuations that were compatible with the deletion patterns of these speakers. Social information and categorization thus shapes how listeners perceive speech. Effects of social information on speech processing may also arise when participants are merely told what the background of a speaker is. Believing that a speaker is from Detroit or Canada [2] can change how listeners label certain vowels. Similarly, believing that a speaker is from Syria or from Portugal can impact social expectations and increase or decrease intelligibility of Arabic-accented German [16].

The focus of this study is on the effect of perceived ethnicity on speech intelligibility and accent ratings. Different studies have reached different conclusions concerning whether social expectations, top-down cues, and biases do or do not lead to processing costs or negative judgments [6,9–12,16–24]. Shifts in speech and speaker perception due to higher-level cognitive states have real-life consequences. Thus, gaining a better understanding of the conditions under which perceived ethnicity modulates speech comprehension and evaluation may help develop tools to reduce stereotyping and inform cognitive models of speech processing. Driven by theoretical and methodological differences in previous research, this study seeks to establish the extent to which effects of perceived ethnicity on speech processing depend on three sources of variability: experimental method, speech context, and age group. These factors are underrepresented in the current literature, with most studies considering results from one type of experimental method (e.g., ratings), one type of population (e.g., university students), and one type of speech context (e.g., standard language) that is not representative of the stylistic diversity in daily language exposure.

There are two frequently debated theoretical frameworks that have led to conflicting results on the effect of perceived ethnicity on speech processing. Bias-based approaches [6,25], such as the reverse-linguistic stereotyping [8,26,27], assume conscious misunderstanding of the speech signal or a decrease in perceptual effort where speakers are classified as nonnative (I use the terms native and nonnative speaker to distinguish between a person who grew up speaking a given local variety and uses it regularly and a person who acquired a foreign language at school age or later. For critical evaluation of the term native speaker, see [28]). According to this account, listeners activate biases or stereotypes about nonnative speakers via previously established language attitudes and ideologies, resulting in perceived phantom accentedness and reduced comprehension. Employing the matched-guise technique [27], [6] found that a photograph of an ethnically Asian American woman presented during playback of a classroom lecture in Standard American English resulted in lower lecture comprehension as measured by cloze test accuracy, more negative accentedness ratings, and lower ratings of teaching competence than the same lecture presented along a photograph of a white American woman. Reductions in speech comprehension scores and accentedness ratings were explained on the basis of stereotypical attributions to nonnative speakers, leading to negative expectations about the speech quality (despite hearing Standard American English) and the speaker herself. Rubin replicated some of his findings in diverse populations, by collecting teachers’ evaluations in a classroom setting [26] and older patients’ ratings of health care assistants [29]. In the latter study, a talker with an American accent was rated as harder to understand, as causing more interference with comprehension, and as sounding less American when presented alongside information (personnel file) and a video-recorded talk of a Latino health care assistant compared to a white assistant. However, the study failed to establish differences in comprehension accuracy, as measured by true-false questions, though participants were more likely to make ‘true’ judgments when they perceived the speaker to be white.

Negative effects of perceived ethnicity on accentedness ratings have been replicated in several studies using Standard American [9], native and nonnative American English [23] as well as American and Indian varieties of English that are usually associated with distinct prestige levels [12]. Further empirical evidence provides mixed findings, showing that bias-based effects on accent ratings vary across presentation contexts such as, for example, the presence versus absence of background noise [19], static pictures versus videos [24], silent video versus audio versus audiovisual clips [17], blocked versus mixed designs [24], between-subject versus within-subject design [24], visual styles and photograph choices [11], and the use of nonnative speech [16,17]. These studies report a lack of ethnicity effects, reversed effects, or effects that are limited to certain conditions (e.g., only under noise, only with audio stimuli).

In contrast, socially indexed exemplar-based accounts rest on the assumption that shifts in speech perception and evaluation result from anticipation of a speaker’s accent based on his/her ethnicity [9,10,17,30]. According to this account, activation of a social category will result in the activation of episodic traces that are consistent with, or linked to, the social category. Seeing an Asian speaker should activate specific foreign-accented speech expectations and enhance speech intelligibility. Speech processing should be enhanced because the accent that is stereotypically associated with a speaker is congruent with the ethnicity attributed to the speaker (it should be pointed out that such a prediction would be in line with other accounts too, such as the rational exemplar-based model or the ideal adapter framework [31–33]). According to this theory, Rubin’s findings simply reflect matching expectations rather than biases or stereotypes. For example, [10] tested American university students (native speakers of English) and found Chinese foreign-accented speech embedded in noise to be more intelligible (as measured by a sentence-repetition task) when a photograph of the ostensible talker showed a Chinese face than when it showed a white European face or a silhouette. According to [10], this enhanced comprehension, driven by socioindexical cues, provides evidence against reverse-linguistic stereotyping. However, it should be noted that [10] analyzed transcription accuracy only for the final word in an utterance to examine effects of semantic predictability, and did not assess accentedness ratings or cloze test accuracy in a manner, which would have enabled direct comparison with the work of Rubin. This methodological disparity implies that a direct evaluation of the reverse-linguistic stereotyping account is unwarranted.

Similarly, testing university students in the multicultural Canadian city of Vancouver, [9] showed that native Canadian English in noise was more intelligible when participants were presented with a photograph of a white Canadian than when presented with a Chinese Canadian, as measured by a sentence-repetition task. The authors also collected accentedness ratings and found that photographs of Chinese Canadians were associated with higher accentedness ratings than photographs of white Canadians. While the rating results are compatible with bias-based accounts, the intelligibility enhancements are not, because racial attitudes did not predict listener performance. Instead, lower intelligibility for Asian primes were explained on the basis of a mismatch between the social expectations triggered by the Asian ethnicity and the unexpected standard speech. The authors interpreted their findings within a framework that links social and linguistic representations and weights certain linguistic representations over others [30]. Following this account, seeing a Chinese Canadian should activate representations of Chinese-accented speech and, if the speech signal does not match with the expected accented speech, intelligibility of standard Canadian speech is decreased. Empirical support for such an account is manifold, and shows that social predictions can result from a listener’s experience with certain speakers and their language use [4,7,17,18,31,33].

It remains unclear whether the apparent paradox in research findings and theoretical contradictions is a likely consequence of methodological choices that tap into distinct aspects of social processing. The bias-based accounts frequently make use of subjective measures such as ratings and evaluative judgments, and at times cloze test accuracy. Rating tasks elicit explicit assessments of a speaker’s social characteristics to make claims about how they induce perception of different social attributions such as ethnicity [34] or degree of accentedness [35]. Ratings are modulated by post-perceptual decisions and participants are usually not under time pressure; they have more control over their judgments, and are more likely to rely on introspection, with the resulting measure assessing interpretation of speech on the decision level rather than perception of speech [24]. Driven by the extent to which a social trait is perceived, the task taps into listeners’ subjective beliefs about how accented the speech appears to be. The associations that people have between a certain speaker and a specific way of talking are, therefore, likely to reveal post-perceptual decision biases and stereotypes.

Researchers addressing ethnicity effects within exemplar-based accounts usually employ implicit measures, such as the sentence-repetition task [9,10], although recent evidence in favor of these accounts comes from accent ratings too [17]. The sentence-repetition task assesses the degree to which an utterance has been understood by measuring participants’ accuracy when asked to transcribe an utterance in standard orthography [36]. In their seminal paper, [36] showed that accentedness and speech intelligibility are clearly distinct dimensions in the assessments of speech, despite their weak correlation. A strong foreign accent does not necessarily result in reduced speech intelligibility. This suggests an incompatibility between different assessments. The repetition task can provide an answer to the question of whether socially associated primes, such as perceived ethnicity, influence the way speech is understood and interpreted. While the task is usually performed in the absence of time restrictions, it does demand attention, in order to recall and repeat or transcribe a given utterance. Thus, this behavioral measure taps more directly into comprehension performance, during which a subjective evaluation of a talker’s ethnicity and accent is possible, but not explicitly required. As such, it can shed light on the extent to which stereotypes and social expectations implicitly influence speech comprehension. The explicit measures (such as rating elicitation) and the implicit measures (such as utterance repetition) each allow for the detection of social expectations or stereotypical associations, but in different ways, which has produced the conflicting results familiar from previous studies.

## Current study

The use of a multi-method approach is desirable to integrate findings from diverse perspectives, in order to better understand the effects of perceived ethnicity on speech processing. The theoretical paradox we have seen may be clarified when explicit and implicit methodologies in different speech contexts are compared in a within-subject design. The first aim of this study was to test the predictions of both theoretical frameworks by evaluating ethnicity effects in an explicit task (accentedness ratings) and an implicit task (sentence repetition) across various speech contexts in German (native accent, foreign accent, and regional accent).

The central hypothesis was that listeners alter the way they interpret and evaluate speech as a function of perceived ethnic similarity (same versus different). The extent to which this is accomplished was assessed in two ways. First, we examined whether Asian primes lead to increased or reduced sentence-repetition performance and greater or lesser accentedness ratings, in a manner predicted by the two theories. According to exemplar-based accounts, anticipating a talker’s accent should increase repetition performance and, thereby enhancing speech intelligibility. More specifically, Asian primes should increase the intelligibility of foreign-accented speech, while white European primes should increase the intelligibility of regional and standard speech. Similarly, accentedness ratings should change as a result of varying expectations. In line with the exemplar approach, an interaction between ethnicity and speech context should be detected. Accentedness ratings should be less negative (i.e. rated as having no or a weak accent) for the white European primes compared to the Asian primes in regional and standard speech while the opposite should hold for foreign-accented speech. In contrast, bias-based accounts predict a drop in sentence-repetition performance and more negative accentedness ratings (i.e. rated as having a stronger accent) for the Asian compared to the white European primes in all speech contexts, because voices perceived to be from nonnative speakers activate stereotypes or biases. No interaction between ethnicity and speech context should emerge on this view. Instead, we would anticipate a main effect of ethnicity, with lower sentence-repetition performance and more negative accentedness ratings for an Asian prime compared to a white European prime.

The second aim of the paper is to examine the stability of the ethnicity effect on speech processing by assessing age-related performance across tasks and speech contexts in diverse non-university populations, namely teens, younger adults and older adults. Efficient language comprehension is essential for successful social interaction for both younger and older adults. The acquisition and maintenance of linguistic skills is, however, strongly modulated by experiential, cognitive, and perceptual factors. While certain aspects of cognition might be preserved or even improve as a function of age (e.g., vocabulary, world knowledge, and different aspects of language comprehension; [37–40]), others might undergo negative changes, such as poorer speech perception and word identification [41], as well as increasingly stereotypical inferences [42]. Evidence for the preservation of top-down reliance on the sentence or discourse context in order to comprehend language is mixed (for a recent review, see [43]). When listening to accented speech or to speech in noise, older listeners seem to rely heavily upon context. It has also been argued that many age-related differences identified in behavioral experiments might simply have resulted from weaker responsiveness of older subjects to task demands in unnatural experimental situations [44,45]. More generally, it has been suggested that age should not be seen as “a situation where older individuals simply do the same task more poorly than younger individuals” ([46], p.2), but as a situation where younger and older adults may to some extent recruit different cognitive strategies and neural resources to support a similar level of behavioral performance [40,47,48]. Changes in speaker-dependent processing across age groups are therefore possible.

Alternatively, however, stronger ethnicity effects might arise due to distinct life experiences and generational cultural differences. Driven by social media and globalization, older adults have presumably experienced less diverse sociolinguistic contexts (e.g., educational contexts) compared to teenagers and younger adults. Less diverse social environments and less regular encounters with counter-stereotypical information might contribute to biases [49].

If stereotypical inferences increase with age due to cognitive or/and experiential factors, older adults would be expected to show stronger ethnicity effects in both tasks compared to the other age groups. A three-way interaction of group, ethnicity, and speech context would answer the question about the stability of the ethnicity effect on language processing across different age groups.

Finally, the study set up allowed an exploration of adaptation to speech. Although no a priori predictions were made regarding changes in sentence-repetition performance in light of perceived ethnicity during the course of the experiment, prior studies suggest improvements after brief exposure to accented speech [22,50,51]. Therefore, the statistical analyses for the repetition task included experimental part (first and second) as a fixed effect. The dynamic use of social information linked to linguistic representations can inform theoretical discussions concerning the adjustments to and updating of probabilistic cue distributions across age groups [31,33].

## Method

### Participants

A total of 172 healthy monolingual non-university participants were recruited at high schools, senior clubs, retirement houses, and online job portals. They were made up of 72 teens (mean age 14.1, SD = 0.8, range 12–16, 33 girls, 39 boys), 50 younger adults (mean age 36, SD = 4.5, range 30–45, 33 women, 17 men), and 50 older adults (mean age 77.6, SD = 5.6, range 70–92, 33 women, 17 men). Participants were of white European ethnicity and all grew up speaking German. Both adult groups were approximately matched for educational level. Teens were tested at their respective high school, most adults were tested in the lab, and some older adults were tested in a retirement home. Language disabilities were ruled out via a questionnaire. Only younger and older adults underwent a hearing sensitivity test. Using an Oscilla USB330 audiometer, pure-tone thresholds for octave frequencies from 0.5 to 8 kHz were measured for both ears (air conduction thresholds only). Since the ability to hear high frequencies typically deteriorates with age, individual mean pure-tone average thresholds (PTA) were computed over 0.5, 1, 2, and 4 kHz for the better ear. Younger adults had a significantly lower PTA (6.9, SD = 4.2) than older adults (27.5, SD = 13). Lower PTA indicates better hearing sensitivity. There was no correlation between hearing sensitivity and age for younger adults (r = .19, p >.18), but the correlation was significant for older adults (r = .50, p < .001). There were 13 older adults wearing hearing aids during the experiment. The adult participants received €20 for participation, while teens received cinema or bookstore vouchers. The Ethical Committee of the Albert-Ludwigs-Universität Freiburg approved the study with teens (no 202/16) and the study with adults (no 348/17) individually. Written informed consent was obtained from all adult participants and from the parents of the teen group.

### Design

Using a within-subject design, this study examines performance in a sentence-repetition task and an accent-rating task with photographic visual primes. In the repetition task, participants transcribed or repeated utterances (in three speech contexts) that were presented to them along with a static photograph of a given talker (six photographs). To control for inter-speaker variability in the three speech contexts, one speaker for each accent (Standard German, Korean-accented German, Palatinate regional accent of German) was used [for more details on the recordings, see 52], and two different versions of each recording were created by minor changes to the effective vocal-tract lengths (see also [19,53]). This allowed for a given experimental list to contain each accent paired with each ethnicity (e.g., the foreign-accented talker in voice version 1 was paired with an Asian prime, while the same foreign-accented talker in voice version 2 was paired with a white European prime).

After the repetition task, participants listened to one utterance produced by each talker once and were asked to provide ratings of comprehensibility on a scale from 1 to 7 (with 7 being very poor) and accent strength on a scale from 1 to 5 (where 5 means having a strong accent), as well as to guess the geographic origin of the talker. Each participant heard 36 utterances, with 12 utterances per speech context. Half of each context’s utterances were assigned to an Asian prime (n = 6) and half were assigned to the white European prime (n = 6). Speech contexts and talkers were presented in mixed blocks (similarly to [9]) and a randomized order so that talker and voice versions were distributed equally across the experiment. Six main counterbalanced lists were created; these varied the sets of 12 sentences across the three speech contexts and the two voice versions for each face within each speech context. In addition, in order to balance the two face primes assigned to the sentences within each speech context, we created six additional versions of the main lists, which were otherwise identical.

### Materials and stimuli

**Fig 1:**
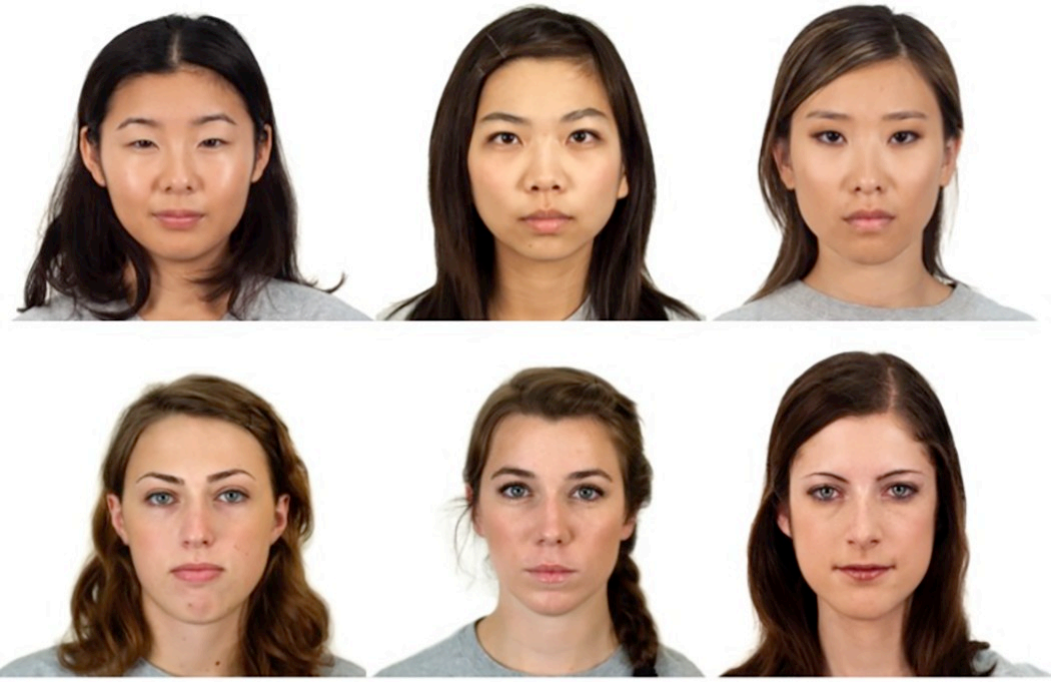
Visual stimuli. Photographs of women of Asian ethnicity and white European ethnicity used in both the rating and sentence repetition tasks.

Visual stimuli were selected from the Chicago Face Database [54] and included six photographs of women with neutral facial expressions, half of which were Asian and the other half of white Caucasian ethnicity (see Fig 1). The women were matched with respect to age, hairstyle, hair length, and ratings of attractiveness and trustworthiness. Acoustic stimuli consisted of 36 short utterances in German, with a total length of 193 words (mean utterance word length 5.3, SD = 0.9; e.g., *Das Bild gibt dem Jungen einen Stuhl* ‘the picture gives the boy a chair’; taken from [52]). These were spoken by three female talkers, each representing one speech context: Standard German, Korean-accented German, and Palatinate German. All utterances were syntactically correct but semantically anomalous, in order to reduce the contribution of context to sentence recall.

Using Praat [55], the f0 and formant dispersion (but not duration or pitch) of the original recordings were changed to obtain two different versions of each voice. The recordings were altered by changing effective vocal-tract lengths (for a similar method, see [19,53]). To achieve this, the f0 of the original utterance was multiplied by a factor of 0.95 (to create voice version 1) and 1.05 (to create voice version 2).

Finally, speech-shaped noise (i.e. noise with a comparable power spectrum to the original speech) was created with PRAAT based on the recordings, and was then combined with the utterances. To obtain speech-shaped noise, a procedure suggested by [56] was applied. A sequence of phases uniformly distributed between 0 and 2pi (*randomUniform (0*, 2 * pi)) was first specified, before creating a new spectrum object with power matching the original sound, but with random phase.

### Procedure

Each participant was tested separately in a quiet room and was seated in a comfortable chair facing a computer monitor. Acoustic stimuli were presented over loudspeakers. Participants were asked to listen to each utterance spoken by the talkers whose faces appeared concurrently on the display. They received the following instructions (translated from German): *We are investigating whom you can understand best in noise*. *You will hear short utterances*. *At the same time*, *you will see a picture of the person who spoke the utterance*. *Each utterance is followed by a beep*. *You can then repeat what you understood*. *For each utterance*, *please pay attention to the picture of the person*, *so that you are able to identify that person later*.

Each participant was presented with a randomized order of one of the experiment lists. A beep tone followed each utterance. Teens were asked to transcribe what they heard on a paper form, while younger and older adults were asked to repeat the utterances (due to age-related changes in the speed of writing for older adults). Oral responses were recorded using a Sennheiser 840S microphone. Participants pressed the space bar to move to the next utterance. Upon completion of the entire sentence-repetition task, participants were again presented with each talker in each speech context, and were asked to provide ratings for each talker. For the purpose of this research, only accent strength and geographic origin were considered. Participants also filled in a background questionnaire prior to testing. Each experimental session consisted of several language production and comprehension experiments, as well as cognitive tests (none of which are reported here because they are part of another project, see [57,58]). An experimental session lasted between 1 hour (teens) and 2.5 hours (younger and older adults).

### Data coding

For the sentence-repetition task, the proportion of all words that participants reported correctly was calculated (both function and content words). Following previous studies [9,52], response deviations from the target word were scored as correct if they a) contained typographical errors (e.g., *Balon*, *Baloon*, *Ballong* for *Ballon* “balloon”), b) represented imitations of accented target words (e.g., *Ente* “duck” produced as the regional variant *Ent* in the regional accent condition, see [52]), or c) were adjusted to match the context with respect to morphosyntactic features, with the word stem otherwise correctly identified (e.g., *Fahnen* “flags” for *Fahne* “flag”, *heirate* “marry” instead of *heiratet* “marries). Two participants from the older adults group were excluded from the final analysis, as their overall accuracy across all speech contexts was lower than 1%. Across the remaining 170 participants, 6118 utterances and 32795 word responses (36 utterances and 193 words per participant) as well as 1008 accent ratings (six ratings per participant) were analyzed. Due to an experimental error, one utterance had to be removed from the final analysis of two teens, and one word had to be removed from the final analysis of three younger adults. For the accent ratings, 12 data points were missing because participants failed to provide ratings.

### Statistical analyses

A linear mixed effect logistic regression model for binary response was fitted to the data (*glmer*, implemented in the R package *lme4*, R Core Team, 2018 [59,60]). Word responses (correct or incorrect) were taken as the dependent variable. Deviation from the mean coding was used, to allow easier interpretation of main effects in the presence of interactions. With deviation from the mean coding, the intercept corresponds to the grand mean, and the coefficients lie on a log-odds scale. A full model was built, as justified by theoretical predictions [61], and included *Accent* (standard, regional, foreign), *Face* (Asian, white European), *Group* (teens, younger and older adults), and *Exp*.*part* (experimental part: first, second) as fixed effects, as well as all possible interaction terms between the fixed factors. Participants and items were modeled with random intercepts. Random slopes for *Accent*, *Face*, and *Exp*.*part* by participants and for *Group* by items were added. The optimizer *optimx* was used to fit the model [62]. The four-way interaction was not significant nor did it improve the model fit. Therefore, it was not considered in the final model. Significant interactions were followed up by post hoc comparisons extracted from the full model using the *emmeans* package [63].

Accent rating data were analyzed using a cumulative link mixed model, this being a linear mixed effect model extended to ordinal data [64]. An ordinal regression approach does not require the assumption of an equal-interval scale. Due to the nonlinearity of the link function, the model estimates cumulative rather than absolute probabilities of observing each category. The default "logit" link was used. The model output plots regression coefficients, which can be transformed to odds ratios by exponentiating the coefficients. Using deviation from the mean coding, the model included ratings as the dependent variable, and *Accent*, *Face*, and *Group* as fixed effects, as well as all interaction terms between these factors. Participants were modeled with random intercepts and random slopes for *Accent* and *Face*. Significant interactions were followed up with post-hoc comparisons, as extracted from the full model using the *emmeans* function.

## Results

### Sentence repetition

**Fig 2:**
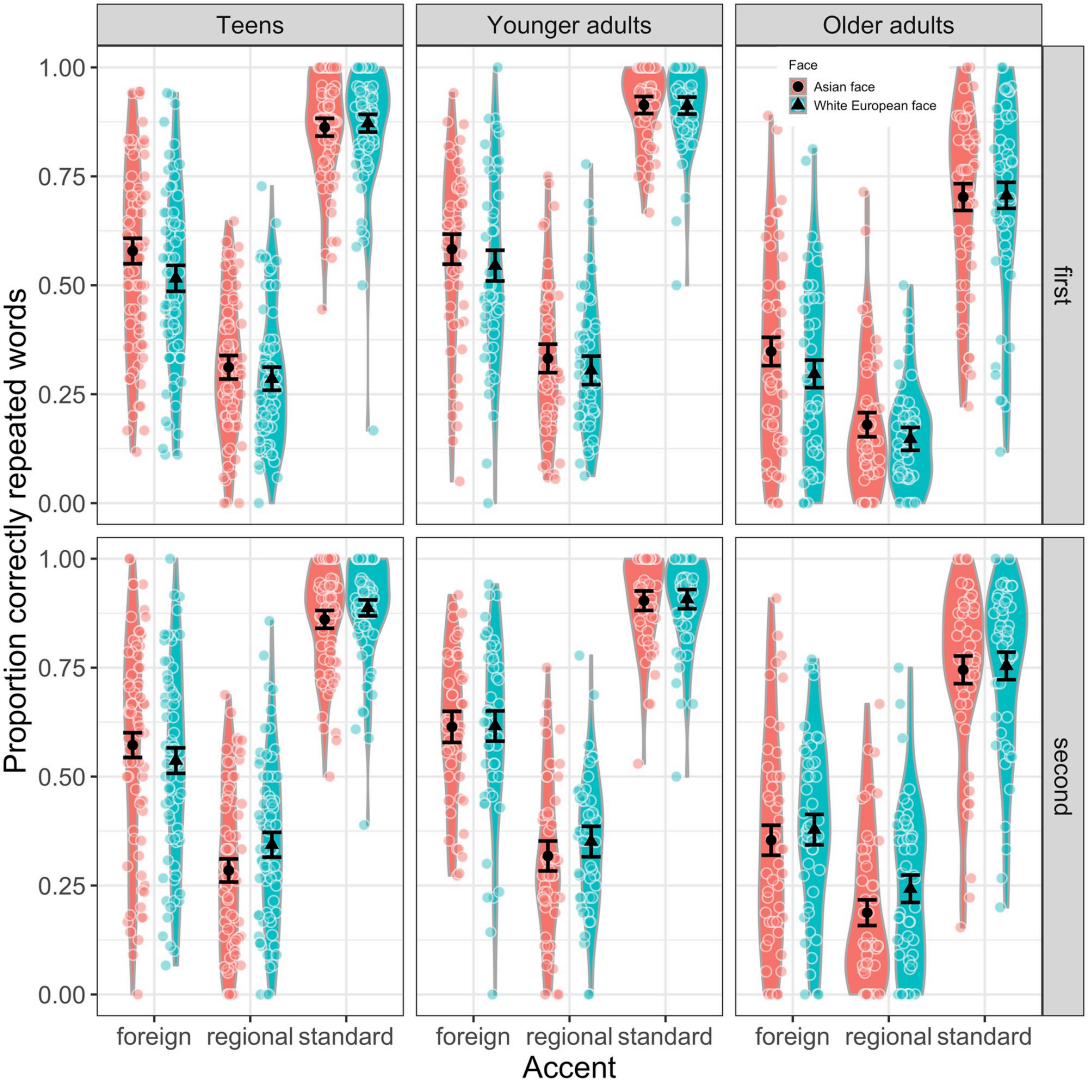
Sentence repetition. Proportion of correctly repeated words in the respective speech context for the first and second half of the experiment, and for each listener group. Black dots represent the overall means and the colored dots show the individual participant means. The violin plots depict probability density, i.e. the wider the shaded area, the more data is located in that area. Error bars represent 95% confidence intervals.

Table 1 shows the proportions of correctly repeated words (raw data) for each speech context and face, and for each age group. As can be seen, the accuracy was overall higher in the foreign accent for Asian primes compared to white European primes, and it was somewhat higher in the regional and standard accent for white European primes compared to the Asian primes. Fig 2 visualizes performance in each condition for the first and second half of the experiment, showing substantial individual variation. The statistical model (see S1 Table) showed that 48% of the variance was explained by both the fixed and random factors (conditional R^2^ = 0.479). The model revealed main effects of *Accent*, *Group*, and *Exp*.*part*, but not *Face* (the intercept was significant [b = 0.264, SE = 0.013, z = 2.040, p = .041]). The regional accent was the least intelligible, i.e., the proportion of correctly repeated words was the lowest [b = -1.439, SE = 0.027, z = -52.786, p < .001]. This was followed by the foreign accent [b = -0.320, SE = 0.025, z = -12.808, p < .001]. The group of younger adults performed best across all speech contexts [b = 0.483, SE = 0.075, z = 6.433, p < .001], followed by the teens [b = 0.235, SE = 0.070, z = 3.358, p < .001]. Repetition performance across all speech contexts was lower in the first half of the experiment than the grand mean [b = -0.051, SE = 0.021, z = -2.431, p = 0.015], suggesting that listeners adapted to the talkers, i.e. repetition performance increased during the course of the experiment.

**Table 1:** Mean repetition accuracy and mean accentedness ratings.

Accent/Face	Teens		Younger adults		Older adults	
	*Repetition*	*Ratings*	*Repetition*	*Ratings*	*Repetition*	*Ratings*
**Foreign**						
Asian	0.58 (.49)	3.61 (.86)	0.60 (.49)	3.61 (.86)	0.35 (.48)	3.18 (1.15)
W. European	0.53 (.50)	3.42 (.96)	0.58 (.49)	3.84 (.82)	0.34 (.47)	2.54 (1.39)
**Regional**						
Asian	0.30 (.46)	3.07 (1.27)	0.33 (.47)	3.18 (1.44)	0.18 (.39)	2.90 (1.43)
W. European	0.31 (.46)	2.96 (1.35)	0.33 (.47)	3.08 (1.45)	0.19 (.40)	2.40 (1.39)
**Standard**						
Asian	0.86 (.35)	1.63 (.78)	0.91 (.29)	1.34 (.63)	0.72 (.45)	1.67 (.83)
W. European	0.88 (.33)	1.31 (.62)	0.91 (.29)	1.20 (.53)	0.73 (.45)	1.47 (.97)

Mean repetition accuracy and mean accentedness ratings for the Asian and white European ethnicity within each group and accent. Standard deviations are given in brackets.

Importantly, while no main effect of Face emerged [b = 0.012, SE = 0.022, z = 0.546, p = 0.585], there was an interaction between accent and face, confirming changes in speech intelligibility (i.e. successful sentence repetition) across speech contexts as a function of perceived ethnicity. The effect was most pronounced in the foreign accent, with increased intelligibility in the Asian guise compared to the white European guise [b = 0.067, SE = 0.0196, z = 3.401, p < .001], as predicted by exemplar-based accounts. There was also a significant interaction with group, showing that the effect of face was more pronounced in the group of teens compared to the other two groups [b = 0.061, SE = 0.026, z = 2.368, p = 0.018]. Perceived ethnicity did not lead to enhanced intelligibility in the regional accent or in the standard accent in the first half of the experiment, but there was a significant interaction with experimental half in the regional context [b = 0.039, SE = 0.020, z = 1.959, p = 0.050]. This interaction suggests an intelligibility enhancement for the white European guise compared to the Asian guise in the second part of the experiment.

To further investigate significant interactions, pairwise contrasts were extracted from the model to assess relationships between speech intelligibility and the fixed factors. The results, presented in Table 2, confirm an effect of ethnicity on intelligibility in the foreign-accented speech and, marginally, in the second half of the regional speech for the group of teens. Older adults showed a marginal effect of ethnicity in the first half of the foreign-accented speech and the second half of the regional speech. Interestingly, no ethnicity effects emerged in the group of younger adults. While the intelligibility enhancements in the foreign accent and the marginal second-half enhancements in the regional accent are in line with the exemplar-based theory, they are relatively weak, and vary as a function of age.

**Table 2:** Pairwise contrasts for sentence repetition accuracy.

Accent	Teens			Younger adults			Older adults		
	*Est*.	*SE*	*z*	*Est*.	*SE*	*z*	*Est*.	*SE*	*z*
**Foreign**									
1^st^ exp. part	0.35	0.10	**3.45** *	0.09	0.11	0.82	0.21	0.12	1.72.
2^nd^ exp. part	0.26	0.10	**2.63** *	0.09	0.12	0.81	-0.06	0.12	-0.50
**Regional**									
1^st^ exp. part	0.11	0.10	1.04	0.09	0.12	0.73	0.23	0.14	1.68.
2^nd^ exp. part	-0.18	0.10	-1.76.	-0.12	0.12	-1.01	-0.25	0.13	-1.87.
**Standard**									
1^st^ exp. part	-0.10	0.13	-0.79	-0.04	0.16	-0.24	0.05	0.12	0.37
2^nd^ exp. part	-0.13	0.13	-0.99	0.19	0.17	0.11	-0.17	0.13	-1.25

Pairwise contrasts for sentence repetition accuracy between Asian and white European ethnicity within each group, accent context, and experimental part. Coefficients (Est.), standard errors (SE), and z-values were extracted from the mixed effect logistic regression model (Tukey-adjusted for multiple comparisons). Positive estimates indicate the extent to which intelligibility was higher for Asian ethnicity than white European ethnicity.

* p < .05,

^.^ p < .1.

### Accentedness ratings

**Fig 3:**
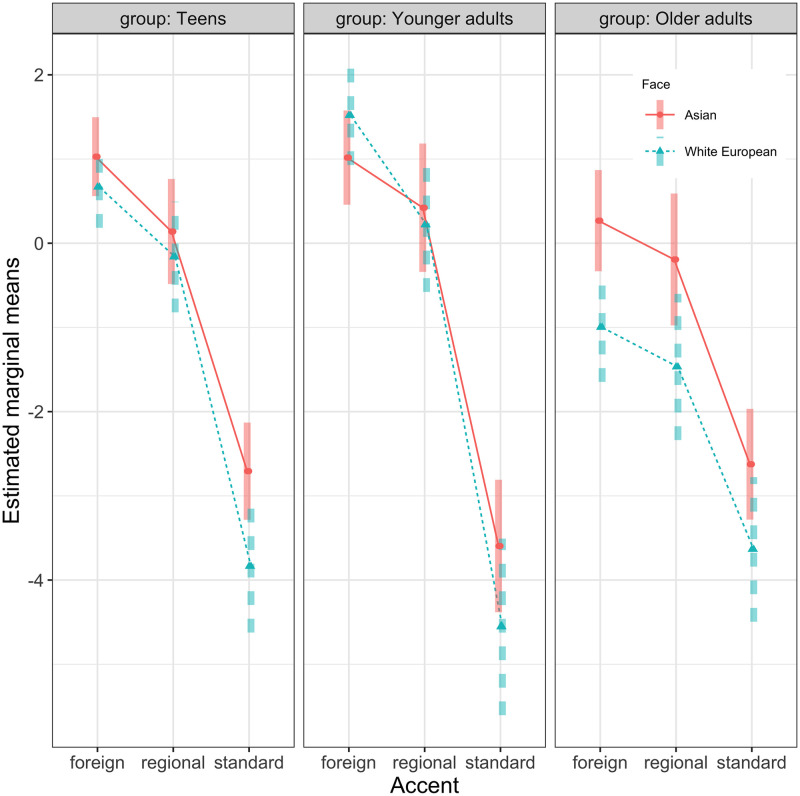
Accent ratings. Estimated marginal means (from the fitted clmm model) for accentedness ratings in the respective speech context and listener group. Error bars represent confidence intervals.

**Fig 4:**
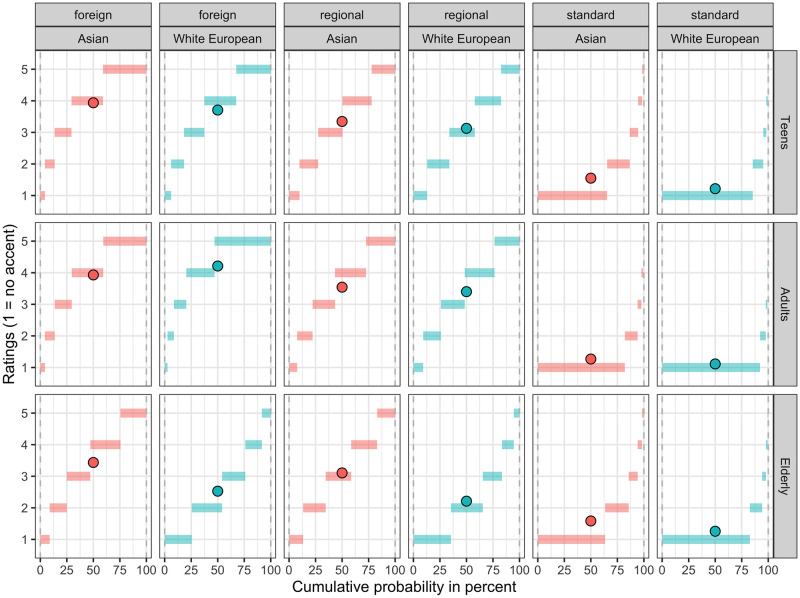
Cumulative probabilities of accent ratings. The length of each horizontal bar represents the modeled probability of a given rating based on the fitted clmm model. The dots represent the predicted values for a given rating in each condition.

Participants’ accentedness ratings in each age group and accent are given in Table 1 (raw data) and Figs 3 and 4 (data from the fitted model). Descriptively, the ratings were higher for the Asian than for white European prime across all groups and accents except with younger adults in the foreign accent condition. The statistical model (see S2 Table) showed that 62% of the variance is explained by both the fixed and random factors (conditional R^2^ = 0.62). The model revealed a main effect of *Face*, such that across all speech contexts, the accent ratings were higher, i.e. more negative, for the Asian ethnicity when compared with the grand mean [b = .334, SE = 0.079, z = 4.241, p < .001], in line with bias-based accounts. There was also a main effect of *Accent*, with foreign-accented speech being the most negatively rated [b = 1.611, SE = 0.134, z = 12.002, p < .001], followed by the regional accent [b = 0.854, SE = 0.135, z = 6.347, p < .001]. There was no main effect of *Group*, but *Group* interacted with *Accent* and *Face*. Compared to the other groups, younger adults’ ratings of the foreign accent were more negative than for the other two accents [b = 0.48574, SE = 0.144, z = 3.369, p < 0.001], and the difference in ratings between the Asian and the white European ethnicity was smaller for the group of younger adults compared to the other two groups [b = -0.223, SE = 0.109, z = -2.045, p = .040]. This shows that the negative effect of the Asian ethnicity on ratings was least pronounced in the group of younger adults. None of the other interactions reached significance.

The overall pattern of results supports previous suggestions in the literature. In line with bias-based accounts, the same acoustic signal was evaluated as more accented in the context of Asian women than white European women. However, differences between groups and across speech contexts emerged. To evaluate the outcome within each group and speech context, pairwise contrasts were extracted from the model. As can be seen in Table 3, older adults rated all speech contexts more negatively when they were associated with photographs of Asian women than photographs of white European women. Teens and younger adults also showed a negative effect of ethnicity on accent ratings, but it was limited to the standard condition, with younger adults showing a marginally significant effect only.

**Table 3:** Pairwise contrasts for accentedness ratings.

Accent	Teens			Younger adults			Older adults		
	*Est*.	*SE*	*z*	*Est*.	*SE*	*z*	*Est*.	*SE*	*z*
Foreign	0.36	0.31	1.16	-0.50	0.38	-1.33	1.27	0.43	2.98*
Regional	0.30	0.34	0.89	0.20	0.42	0.49	1.28	0.45	2.82*
Standard	1.13	0.41	2.74*	0.96	0.57	1.68.	1.01	0.48	2.10*

Pairwise contrasts for accentedness ratings between the Asian and white European ethnicity within each group and accent. Coefficients (Est.), standard errors (SE), and z-values were extracted from the cumulative link mixed model (Tukey adjusted for multiple comparisons).

* p < .05,

^.^ p < .1.

## Discussion

This study examined the hypothesized causal link between perceived ethnic similarity and social expectations in speech evaluation and comprehension, by comparing results from an implicit and an explicit task across three speech contexts and three age groups. There are two major conclusions to be drawn: first, both experimental methods showed partially successful integration of social information, but the consequences for speech processing and evaluation diverged. Sentence repetition performance as a function of perceived ethnicity squares best with socially indexed exemplar-based theories, but this holds largely for the foreign-accented context [9,10,17,30]. Overall accentedness ratings were most consistent with bias-based theories for each speech context [6,65]. Thus, both theoretical frameworks can account for certain of the previous findings, but the diverging results suggest that the theories are associated preferentially with different types of task. Second, the effects varied across age groups and speech contexts, with younger adults showing weak or no effects of ethnicity in either task. In addition, repetition performance varied during the course of the experiment, showing that listeners adjusted to the speech and to the talker. The malleability of ethnicity effects underlines the importance of a substantial scrutiny of diverse methods, contexts, and populations. The implications of these results will be discussed one by one.

Across all speech contexts and groups, the same acoustic signal was rated as more accented when presented alongside with photographs of women of Asian ethnicity than when presented alongside with photographs of white European women. This result lends credibility to bias-based accounts and reverse-linguistic stereotyping [6,65], according to which stereotypical attributions to a speaker whose ethnicity is perceived as being foreign would result in negative ratings irrespective of the speech context. A closer look at the individual age groups, however, revealed the effect to be clearly pronounced in the group of older adults. In the group of teens and younger adults, the effect was relatively weak: it was limited to the standard accent and was only marginally significant in the group of younger adults.

Why might older adults’ accentedness ratings differ from those of teens and younger adults? Naturally, the interpretation of the task as well as the familiarity with rating tasks might differ between participants and result in large variation. In addition, inhibitory skill is one possible factor hypothesized to give rise to differences in stereotypes across the life span [42]. As inhibition continues to decline with increasing age—in addition to poorer speech perception and word identification [41]–it can cause older adults to make more stereotypical judgments of others. It is reasonable to assume that older adults attributed difficulties arising from processing speech in noise to difficulties arising from accentedness associated with perceived foreign ethnicity, resulting in lower ratings for trials associated with Asian women across all speech contexts.

An alternative explanation is that life-long experiences with certain speakers and their language use shape distributional knowledge [4,31,33,37] and contribute to differences across age groups. Since humans accumulate social expectations through experience with spoken language along with visual cues, more regular interactions with people from diverse places of origin and encounters of counter-stereotypical information might facilitate bias reduction [49]. If older adults were raised or spent their formative years in a relatively homogenous society, they may use and hold to their beliefs about groups more than teens and younger adults. Such factors could potentially contribute to the reduced effects in accentedness ratings for the teens and younger adults, whose social environments tend to be more diverse than those of older adults. Based on the present cross-sectional design, it is difficult to quantify generational cultural differences or to assess how social category formation is modulated by inhibition and/or experiential factors across the life span. A full taxonomy of the nuanced social information that leads to language attitudes, talker categorization, and to linguistic expectations at different life stages is yet to be put forth.

The majority of previous studies reported negative ratings for standard speech with faces perceived as foreign (see [6,9,29,65], and, for static pictures only, [24]), as well as for different varieties of English [12], and even for different visual styles among foreign-looking individuals [11]. The present study replicates those findings, with clear ethnicity effects for the older adult group in the three speech contexts, and an effect in the standard context in the group of teens, as well as a marginal effect in the group of younger adults. However, no effect emerged in the foreign and regional-accented speech in teen or younger adult groups. It appears that, in contrast to the standard accent, the speaker’s origin was easily identified based on the foreign and regional accent, and that the visual information did not add much to the evaluation of the accent. This stands in contrast to both theoretical accounts and to certain previous studies [17].

One possible interpretation of the rating result follows the idea of distinct reliance on visual cues in native versus nonnative speech, as reported by [23]. [23] found that facial cues (white European versus Asian) reduced visual benefits in nonnative speech compared to native speech. Korean nonnative talkers received more negative accent ratings in a short video compared to audio only, while Caucasian English native talkers were rated as less accented in a short video as compared to audio. According to [23], listeners are less able to benefit from visual cues in nonnative speech, without necessarily reducing their reliance on visual cues. Such reduced benefits may equally apply to regional accents.

However, reliance on visual cues in static photographs might produce different effects than seeing the actual person talking. [24] replicated the increased accentedness ratings of native speech for an Asian face with static pictures, but not when speech and face were integrated audiovisually, suggesting that demand characteristics produced by pictures are larger than by videos. The extent to which demand characteristics of pictures might explain the differences across age groups and speech contexts is still in question. Do demand characteristics (along with cognitive and attentional demands in foreign and regional accents) increase with age, resulting in stronger reliance on visual cues?

Finally, it could be that variation in social categorization (and familiarity with a specific accent) resulted in intra- and intergroup variation in language attitudes [1]. Once foreign or regional accents become salient as social categories, listeners activate stereotypes or attitudes, which may or may not be influenced by the additional social category evoked by the static picture. Research on persona construal and social categorization suggests that these social categories are not stable constructs; rather they change across contexts and the life span [66], reinforcing the importance of future studies that incorporate a life span perspective.

A clearly different pattern of results emerged for the sentence-repetition task. Repetition performance with foreign-accented speech increased in the context of the Asian ethnicity compared to the white European ethnicity, albeit only in the group of teens, and marginally in the group of older adults. In contrast, the regional accent was more intelligible (i.e. utterances were better recalled) when it was associated with a photograph of a white European woman compared to a photograph of an Asian woman, but this effect was relatively small, and the marginal effects were limited to the second half of the experiment for the groups of teens and older adults. While this result goes in the predicted direction of socially indexed exemplar-based accounts, the effect is clearly malleable and somewhat ephemeral. The effects may also be explained by other accounts, such as the rational expectation-based account [32] or by the ideal adapter framework [31,33]. Both approaches model the degree of expectation and adaptation through Bayesian belief update. The successful comprehension of noisy speech input depends on several probabilistic factors that can change for any given context and speaker and be more or less informative. In addition to prior knowledge and generalizations to other situations and speakers, listeners adjust their expectations based on newly learned cue distributions in the speech input of a specific talker and for a specific task. Such a framework might thus provide a potential explanation for the habituation effects in the present study.

An interesting aspect of the regional condition is its relatively low intelligibility; and the questionnaire results confirmed the difficulties associated with its identification as a regional dialect of German. Several participants assumed that they were listening to nonnative speech. For trials associated with white European women, the geographic origin of a talker speaking a regional dialect of German was less likely to be assessed as German, Swiss or Austrian than when speaking Standard German (teens: 65% vs. 96%, younger adults: 60% vs. 92%, older adults 58% vs. 88%). Similarly, for trials associated with Asian women, a talker of a regional dialect of German was less likely to be assessed as German, Swiss or Austrian than when speaking Standard German (teens: 18% vs. 40%, younger adults: 16% vs. 34%, older adults 36% vs. 72%). As a consequence, listeners might have needed more time to adjust to the regional speech and to update probabilistic cues and expectations accordingly. This flexible categorization of speech from nonnative to native and the resulting adjustment of expectations within the course of an experiment represent an interesting future testbed for the effects of ethnicity on speech adaptation.

No effect of ethnicity on intelligibility of the standard accent emerged (note that accent ratings showed a reliable ethnicity effect in this condition), in contrast to reports from previous studies [9]. This is surprising given the longstanding research reporting significant improvements in word recognition and speech perception under noise when an unintelligible speech signal is complemented with visual cues as an additional source of information [67–69]. In the regional and foreign accents, reliance on the visual cues was clearly useful when making sense of apparently unclear or noisy speech. Given the relative ease with which the standard accent was comprehended, it seems reasonable to assume that the speech signal carried more weight for listeners than did the visual cues. In addition, repeated personal experiences with distinct ethnicities that speak German as their native language within the German cultural context may reduce implicit associations from perceived foreignness to perceived accentedness [70]. While it is not unusual to meet native speakers of German with diverse migration backgrounds in present-day Germany, it is less likely to meet an Asian person with native or nativelike mastery of the Palatinate German regional variety. As a result, considerable intra- and inter-individual variation as a function of experience should not be surprising. Future research could examine how changing the social environment by including talkers with diverse accents and speech styles may potentially be more effective in reducing biases and expectations than including only a single speech style or ethnicity.

It is also possible that ethnicity effects in standard speech are mitigated when native and nonnative talkers are presented in a mixed fashion, and might appear more clearly in study designs that are limited to one speech context (as in [9]) or to between-subject designs (as in [6,8,24,29]). Methodological differences between studies can certainly yield conflicting findings, and this is true of both implicit and explicit tasks. As pointed out by several researchers, presentation modality (still pictures or videos, blocked or mixed presentation) and cue order (seeing a speaker first or listening first before seeing the face) can reverse ethnicity effects on accentedness ratings [24] or even hirability evaluations [65]. Such variability in findings may lead us to question whether general claims about ethnicity effects are tenable at all (see also [13]).

Note that the present study also employs slightly different measures in the sentence-repetition task for teens than for younger and older adults (transcription versus oral response). This age-related adjustment to the design was made after the data collection with teens was completed. It is possible that the modality of the task could have influenced the overall effect size, because transcriptions are cognitively demanding and need more time than auditory repetition, which allows more time for the possible activation of attitudes and stereotypes. Interestingly, the main results are very similar across groups—for all groups, the intelligibility of the standard accent is better than that of the foreign accent, with both more intelligible than the regional accent. The main difference between the processing and the rating task with respect to the perceived ethnicity also remains the same, irrespective of the task modality. It would be of use for future methodological studies directly to assess the effect size of ethnicity in transcription tasks compared to oral response tasks.

Despite descriptive similarities with respect to the ethnicity effects on the sentence-repetition performance across the three age groups, clear differences emerged regarding the habituation effect and the size of ethnicity effects. Age-dependent reliance on visual cues underscores diverging demands posed by the listening task, with different consequences for social cues integration across different age groups. There are at least three possible factors contributing to this variability, which need further examination in future studies: experience, cognitive and perceptual demands, and study design (as discussed above). Prior research has demonstrated a disadvantage for visual and auditory speech processing with increasing age. [71] suggest that, while word recognition generally improves if the talker is visible, listening effort increases as well. By adding background noise to a listening condition, cognitive and attentional demands increase (for a review, see [72]), even more so when listeners must compensate for poorer speech perception and word recognition skills, as associated with aging [41]. Greater cognitive resources may aid in completing a sentence-repetition task under noise [71,73]. Given the general decline in cognitive resources with increasing age, younger and older participants may recruit different cognitive strategies to support a similar level of behavioral performance [40,47,48]. In addition, the lack of ethnicity effects for the younger adults possibly reflects more diverse experiences. Coming from a very broad spectrum of occupations, the variety of human characteristics they experience on daily basis might vary substantially for a kindergarten teacher, a househusband, or an architect.

Finally, both experimental methods show partially successful integration of social information, but the conclusions diverge. A disconnect between linguistic measures and a non-equivalence of sentence repetition and indexical judgments such as accentedness ratings became apparent. It seems clear that these do not to tap into the same underlying construct. Notwithstanding that this is not a novel finding (see [9,36]), the undifferentiated use of implicit and explicit measures across studies continues to retard theoretical progress. [74] raises this important issue and sees a lack of integration between psychometrics and experimental research. He points out (p. 428) that "measurement does not consist of finding the right observed score to *substitute* for a theoretical attribute, but of devising a model structure to *relate* an observable to a theoretical attribute". Measurement is essentially continuous with theory construction; it is therefore important to understand how theoretical attributes (e.g. perceived ethnicity) relate to observations (e.g. as measured by sentence repetition or by accent ratings). The present work contributes to this debate by providing a methodological perspective on ethnicity effects in diverse speech contexts, across three age groups. The malleability of ethnicity effects for both measures shows the importance of substantial scrutiny of the methodological disparities used to support different theoretical accounts, and clarifies the need for further research to address the stability of social effects across intersecting categories, diverse contexts and age groups (see also [11,13,66]).

In summary, the results show that social and linguistic information interact across age groups in more complex ways than previously thought, underscoring the importance of diverse methodological approaches in diverse populations and speech contexts. Even though listeners might benefit from socio-indexical inferences during speech comprehension, successful comprehension is not limited to such inferences, and faces do not always speak volumes. Whether benefits or biases of social inferences for speech processing occur depends on the context in which the social information occurs, how it is perceived and interpreted by individual listeners, and how the experimenter assesses them.

## Supporting information

S1 Table Summary of the mixed effect model for the sentence repetition performance (the intercept represents the grand mean). (DOCX)

S2 Table Summary of the cumulative link mixed model for the accentedness ratings. (DOCX)
