# Supplementary material for: Do faces speak volumes? Social expectations in speech comprehension and evaluation across three age groups
Source: PLoS One. 2021 Oct 28;16(10):e0259230. doi: 10.1371/journal.pone.0259230 (PMC8553087; doi:10.1371/journal.pone.0259230)
Supplement: S1 Table — (DOCX) [file pone.0259230.s001.docx]

**Table S1. Summary of the mixed effect model for the sentence repetition performance (the intercept represents the grand mean).**

|  | Estimate | Std. Error | z | p-value |
| --- | --- | --- | --- | --- |
| (Intercept) | 0.26 | 0.13 | 2.04 | **0.041** |
| Voice[foreign] | -0.32 | 0.02 | -12.81 | **<0.001** |
| Voice[regional] | -1.44 | 0.03 | -52.79 | **<0.001** |
| Face[Asian face] | 0.01 | 0.02 | 0.55 | 0.585 |
| group[Teens] | 0.24 | 0.07 | 3.36 | **0.001** |
| group[Younger adults] | 0.48 | 0.08 | 6.43 | **<0.001** |
| exp.part[first] | -0.05 | 0.02 | -2.43 | **0.015** |
| Voice[foreign] : Face[Asian face] | 0.07 | 0.02 | 3.40 | **0.001** |
| Voice[regional] : Face[Asian face] | -0.02 | 0.02 | -1.13 | 0.257 |
| Voice[foreign] : group[Teens] | 0.03 | 0.03 | 0.84 | 0.404 |
| Voice[regional] : group[Teens] | 0.00 | 0.04 | 0.01 | 0.989 |
| Voice[foreign] : group[Younger adults] | 0.00 | 0.04 | 0.04 | 0.968 |
| Voice[regional] : group[Younger adults] | -0.16 | 0.04 | -4.19 | **<0.001** |
| Voice[foreign] : exp.part[first] | -0.01 | 0.02 | -0.57 | 0.571 |
| Voice[regional] : exp.part[first] | 0.02 | 0.02 | 1.03 | 0.303 |
| Face[Asian face] : group[Teens] | 0.01 | 0.03 | 0.44 | 0.660 |
| Face[Asian face] : group[Younger adults] | -0.00 | 0.03 | -0.04 | 0.968 |
| Face[Asian face] : exp.part[first] | 0.04 | 0.01 | 2.85 | **0.004** |
| group[Teens] : exp.part[first] | 0.03 | 0.03 | 1.13 | 0.258 |
| group[Younger adults] : exp.part[first] | 0.04 | 0.03 | 1.42 | 0.157 |
| Voice[foreign] : Face[Asian face] : group[Teens] | 0.06 | 0.03 | 2.37 | **0.018** |
| Voice[regional] : Face[Asian face] : group[Teens] | -0.02 | 0.03 | -0.79 | 0.429 |
| Voice[foreign] : Face[Asian face] : group[Younger adults] | -0.03 | 0.03 | -1.07 | 0.284 |
| Voice[regional] : Face[Asian face] : group[Younger adults] | 0.00 | 0.03 | 0.12 | 0.907 |
| Voice[foreign] : Face[Asian face] : exp.part[first] | -0.01 | 0.02 | -0.67 | 0.505 |
| Voice[regional] : Face[Asian face] : exp.part[first] | 0.04 | 0.02 | 1.96 | **0.050** |
| Voice[foreign] : group[Teens] : exp.part[first] | 0.02 | 0.03 | 0.68 | 0.495 |
| Voice[regional] : group[Teens] : exp.part[first] | 0.01 | 0.03 | 0.48 | 0.631 |
| Voice[foreign] : group[Younger adults] : exp.part[first] | -0.07 | 0.03 | -2.34 | **0.019** |
| Voice[regional] : group[Younger adults] : exp.part[first] | 0.02 | 0.03 | 0.55 | 0.580 |
| Face[Asian face] : group[Teens] : exp.part[first] | -0.01 | 0.02 | -0.44 | 0.657 |
| Face[Asian face] : group[Younger adults] : exp.part[first] | -0.03 | 0.02 | -1.42 | 0.154 |
| Observations | 32795 | | | |
| Marginal R^2^ / Conditional R^2^ | 0.319 / 0.479 | | | |
