# Supplementary material for: Do faces speak volumes? Social expectations in speech comprehension and evaluation across three age groups
Source: PLoS One. 2021 Oct 28;16(10):e0259230. doi: 10.1371/journal.pone.0259230 (PMC8553087; doi:10.1371/journal.pone.0259230)
Supplement: S2 Table — (DOCX) [file pone.0259230.s002.docx]

**Table S2. Summary of the cumulative link mixed model for the accentedness ratings.**

|  | Estimate | Std. Error | z | p-value |
| --- | --- | --- | --- | --- |
| Face[Asian] | 0.33 | 0.08 | 4.24 | **<0.001** |
| variante[foreign] | 1.61 | 0.13 | 12.00 | **<0.001** |
| variante[regional] | 0.85 | 0.13 | 6.35 | **<0.001** |
| group[Teens] | 0.22 | 0.14 | 1.58 | 0.114 |
| group[Younger adults] | 0.20 | 0.15 | 1.30 | 0.193 |
| Face[Asian] : variante[foreign] | -0.15 | 0.09 | -1.56 | 0.119 |
| Face[Asian] : variante[regional] | -0.04 | 0.10 | -0.39 | 0.700 |
| Face[Asian] : group[Teens] | -0.03 | 0.10 | -0.36 | 0.716 |
| Face[Asian] : group[Younger adults] | -0.22 | 0.11 | -2.05 | **0.040** |
| variante[foreign] : group[Teens] | 0.05 | 0.13 | 0.38 | 0.704 |
| variante[regional] : group[Teens] | -0.05 | 0.16 | -0.33 | 0.742 |
| variante[foreign] : group[Younger adults] | 0.49 | 0.14 | 3.37 | **0.001** |
| variante[regional] : group[Younger adults] | 0.30 | 0.18 | 1.63 | 0.102 |
| Face[Asian] : variante[foreign] : group[Teens] | 0.03 | 0.12 | 0.24 | 0.814 |
| Face[Asian] : variante[regional] : group[Teens] | -0.11 | 0.12 | -0.90 | 0.366 |
| Face[Asian] : variante[foreign] : group[Younger adults] | -0.21 | 0.13 | -1.64 | 0.101 |
| Face[Asian] : variante[regional] : group[Younger adults] | -0.03 | 0.14 | 0.21 | 0.837 |
| Observations | 1008 | | | |
| Marginal R^2^ / Conditional R^2^ | 0.417 / 0.621 | | | |
